# Ginsenoside Rd Attenuates Myocardial Ischemia/Reperfusion Injury via Akt/GSK-3β Signaling and Inhibition of the Mitochondria-Dependent Apoptotic Pathway

**DOI:** 10.1371/journal.pone.0070956

**Published:** 2013-08-16

**Authors:** Yang Wang, Xu Li, Xiaoliang Wang, Waynebond Lau, Yajing Wang, Yuan Xing, Xing Zhang, Xinliang Ma, Feng Gao

**Affiliations:** 1 Department of Physiology, Fourth Military Medical University, Xi'an, China; 2 Department of Emergency Medicine, Thomas Jefferson University, Philadelphia, Pennsylvania, United States of America; King's College London, University of London, United Kingdom

## Abstract

Evidence suggests Ginsenoside Rd (GSRd), a biologically active extract from the medical plant Panax Ginseng, exerts antioxidant effect, decreasing reactive oxygen species (ROS) formation. Current study determined the effect of GSRd on myocardial ischemia/reperfusion (MI/R) injury (a pathological condition where ROS production is significantly increased) and investigated the underlying mechanisms. The current study utilized an in vivo rat model of MI/R injury and an in vitro neonatal rat cardiomyocyte (NRC) model of simulated ischemia/reperfusion (SI/R) injury. Infarct size was measured by Evans blue/TTC double staining. NRC injury was determined by MTT and lactate dehydrogenase (LDH) leakage assay. ROS accumulation and apoptosis were assessed by flow cytometry. Mitochondrial membrane potential (MMP) was determined by 5, 5′, 6, 6′-tetrachloro-1, 1′, 3, 3′-tetrathylbenzimidazol carbocyanine iodide (JC-1). Cytosolic translocation of mitochondrial cytochrome c and expression of caspase-9, caspase-3, Bcl-2 family proteins, and phosphorylated Akt and GSK-3β were determined by western blot. Pretreatment with GSRd (50 mg/kg) significantly augmented rat cardiac function, as evidenced by increased left ventricular ejection fraction (LVEF) and ±d*P*/d*t*. GSRd reduced myocardial infarct size, apoptotic cell death, and blood creatine kinase/lactate dehydrogenase levels after MI/R. In NRCs, GSRd (10 µM) inhibited SI/R-induced ROS generation (*P*<0.01), decreased cellular apoptosis, stabilized the mitochondrial membrane potential (MMP), and attenuated cytosolic translocation of mitochondrial cytochrome c. GSRd inhibited activation of caspase-9 and caspase-3, increased the phosphorylated Akt and GSK-3β, and increased the Bcl-2/Bax ratio. Together, these data demonstrate GSRd mediated cardioprotective effect against MI/R–induced apoptosis via a mitochondrial-dependent apoptotic pathway.

## Introduction

**Figure 1:**
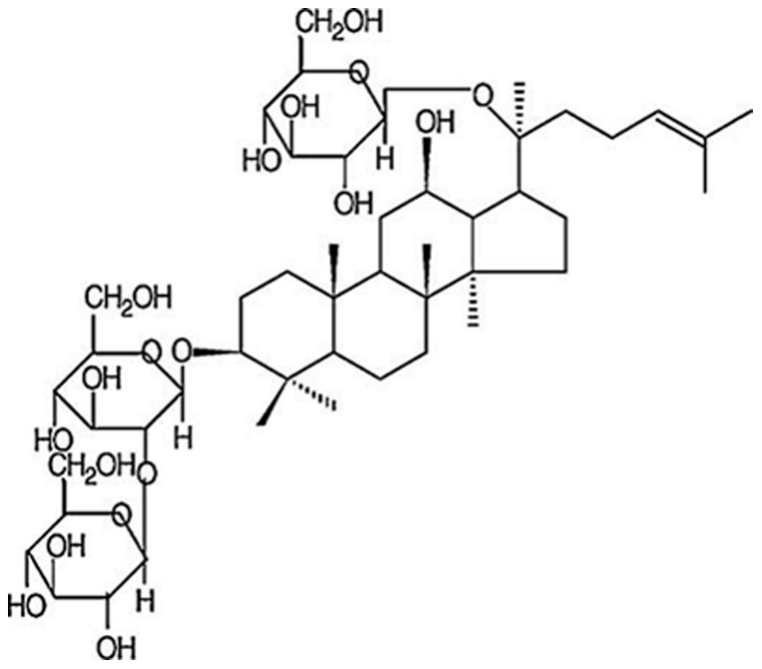
The chemical structure of GSRd. The molecular formula of GSRd (C_48_H_82_O_18_·3H_2_O). Its molecular weight is 1001.

Ginseng, the root of Panax ginseng C.A. Mayer (Araliaceae), is a popular traditional Chinese medicinal herb. Although the mechanisms responsible for ginseng's various effects remain largely unknown, several active ingredients termed ginsenosides have been isolated from the plant [1]–[3]. Ginsenoside Rd, Dammer-24(25)-ene-3β, 12β, 20(S)- triol-(20-O-β-D-glucopyranosyl)-3-O-β-D-glucopyranosyl-(1→2)-β-D-gluco-pyranoside (GSRd, C_48_H_82_O_18_·3H_2_O, molecular weight 1001, Figure 1), one of the major P. ginseng isolates, scavenges free radicals [4], [5], inhibits Ca^2+^-influx via receptor and store-operated Ca^2+^ channels [6], and protects against neuronal apoptosis [4], [7]. Therefore, in addition to being highly lipophilic and capable of easily diffusing across biological membranes, GSRd may have significant advantageous cardiac effects. However, it has not been investigated whether GSRd exerts protective effect against myocardial ischemia- reperfusion (MI/R) injury, or by what potential mechanisms.

Toxic reactive oxygen species (ROS) generated during MI/R both directly and indirectly affect cardiomyocyte function, promoting apoptosis and necrosis [8]. Mitochondria are both a major endogenous source and target of ROS, including superoxide anions, hydrogen peroxide, peroxynitrite, and hydroxyl radicals. Mitochondrial dysfunction increases ROS production, exacerbating oxidant-induced apoptosis [9], [10]. During early reperfusion, ROS burst alters intracellular redox states, modifies the inner mitochondrial membrane potential (MMP), and releases mitochondrial-cytochrome c into the cytosol, ultimately activating caspase-3 in the final apoptotic pathway [11], [12]. Preventing ROS production and preserving mitochondrial integrity are therefore protective against MI/R injury. Clinical evidence demonstrates GSRd potently suppresses ROS generation [4], [13]. It remains unknown whether GSRd may decrease MI/R-induced ROS generation, or whether GSRd may inhibit the mitochondrial-dependent apoptotic pathway.

The phosphatidylinositol-3-kinase (PI3K)/Akt pathway is cardioprotective against MI/R injury [14]–[16]. Additionally, PI3K/Akt pathway activation attenuates mitochondria-mediated apoptosis [17], [18]. Serine/threonine kinase Akt is a primary mediator of the downstream effects of phosphatidylinositol-3 kinase (PI3K), preserving mitochondrial integrity by phosphorylating molecules such as the Bcl-2 family and GSK-3β [19]. Glycogen synthase kinase 3β (GSK-3β) is a serine/threonine kinase; phosphorylated GSK-3β is cardioprotective against MI/R injury [20]. Although GSRd has been demonstrated to be anti-apoptotic by activating PI3K/Akt [21], whether GSRd suppresses mitochondrial-dependent apoptosis during MI/R via PI3K/Akt/GSK-3β signaling remains unknown.

Therefore, the aims of this study were: 1) to determine whether GSRd exerts any cardioprotective effect against MI/R injury; 2) to determine whether GSRd may decrease oxidative stress in rats subjected to MI/R; and if so, 3) to investigate the responsible underlying mechanisms.

## Materials and Methods

### Animals and reagents

This study was performed in adherence with the National Institutes of Health Guidelines for the Use of Laboratory Animals, and was approved by the Fourth Military Medical University Committee on Animal Care. Male Sprague-Dawley (SD) rats weighing 270–320 g were provided by the Experimental Animal Center of the Fourth Military Medical University (Xi'an, China). All animals were allowed free access to food and water, and were maintained at 22–24°C under a 12 hour:12 hour light-dark cycle. GSRd (purity 98%, Tai-He Biopharmaceutical Co. Ltd, Guangzhou, China) stock solutions were prepared in saline containing 10% 1, 3-propanediol (v/v). Fetal bovine serum (FBS) and Dulbecco's modified Eagle's medium (DMEM) were from Gibco (Grand Island, NY, USA). TUNEL apoptosis kit was from Roche Diagnostics (Mannheim, Germany). Propidium iodide (PI), Annexin-V, 3-(4,5-dimethylthiazol-2-yl)-2,5-diphenyltetrazolium bromide (MTT), 2′,7′-dichorofluoresceine diacetate (DCFH-DA), and 5,5′,6,6′-tetrachloro-1,1′,3,3′-tetraethyl-benzimidazol-carbocyanine iodide (JC-1) were from Sigma-Aldrich Inc. (St. Louis, MO, USA). Antibodies against Bcl-2, Bax, caspase3, caspase9, cytochrome c, and β-actin were from Santa Cruz Biotechnology (Santa Cruz, CA, USA). Antibodies against Akt, phospho-Akt (Ser473), GSK-3β, and phosphor-GSK- 3β (Ser9) were from Cell Signaling Technology (Beverly, MA, USA). All other reagents were of standard biochemical quality from commercial suppliers.

### Myocardial ischemia/reperfusion (MI/R) model in rats

Adult male Sprague-Dawley rats were fasted overnight, and anesthetized via intraperitoneal (IP) administration of 50 mg/kg pentobarbital sodium. A micro-catheter was inserted into the left ventricle through the right carotid artery to measure the left ventricular pressure. Myocardial ischemia was produced after exteriorizing the heart via a left thoracic incision, and placing a 6–0 silk slipknot suture around the left anterior descending coronary artery approximately 2–3 mm from its origin. Ischemia was monitored and confirmed by ST segment elevation upon electrocardiogram (ECG). After 30 minutes ischemia, the slipknot was released, and myocardial reperfusion for 3 hours. Rats were randomly assigned to one of the following treatments (n = 8/group): 1) Sham group, receiving vehicle IP injection (10 ml/kg saline) and operative procedures without coronary slipknot; 2) MI/R group, receiving vehicle IP injection (10 ml/kg saline) 30 minutes prior to coronary I/R; and 3) MI/R+GSRd group, receiving GSRd IP injection (50 mg/kg) 30 minutes prior to coronary I/R, a dose established from prior investigations [13], [22].

### Isolation of primary neonatal rat cardiomyocytes and simulated ischemia/reperfusion (SI/R)

Neonatal rat cardiomyocytes (NRCs) were isolated from 1–2 day old Sprague-Dawley rats. Briefly, excised hearts were washed in Hanks balanced salt solution (HBSS; Ca^2+^-Mg^2+^free). Ventricles were freed of associated tissues, minced, subjected to 5–6 0.125% trypsin washes (37°C), filtered, and centrifuged at 1,000 rpm for 10 minutes. Supernatant was resuspended in DMEM containing 20% fetal bovine serum, penicillin (100 U/mL), and streptomycin (100 U/mL). Resuspended cells were placed in a petri dish in a humidified incubator (5% CO_2_, 37°C) for 90 minutes to promote dish surface attachment of non-myocytes suspended in solution. Cells were harvested and seeded onto 60-mm culture dishes. 5-Bromo-2′-deoxyuridine (100 µM) was added during the first 48 hours to inhibit non-myocyte proliferation. Simulated I/R (SI/R) was employed as previously described [23]. Briefly, simulated ischemia buffer (composition in mM: NaCl 98.5, KCl 10, MgSO_4_ 1.2, CaCl_2_ 1.0, HEPES 20, sodium lactate 40, pH 6.8) and simulated reoxygenation buffer (composition in mM: NaH_2_PO_4_ 0.9, NaHCO_3_ 20.0, CaCl_2_ 1.0, MgSO_4_ 1.2, HEPES 20.0, NaCl 129.5, KCl 5.0, glucose 5.5, pH 7.4) were prepared in advance. Confluent-beating cells in 6-well plates were subjected to medium replacement with simulated ischemia buffer, incubated in a hypoxic chamber (of humidified atmosphere 5% CO_2_/0% O_2_ balanced with N_2_ at 37°C) for 3 hours, and then reoxygenated in a standard incubator for 2 hours with medium replacement with re-oxygenation buffer. Cells subjected to control conditions were cultured with normal Tyrode solution (pH 7.4) in a humidified atmosphere of 5% CO_2_/21% O_2_ balanced with N_2_ at 37°C for 5 hours. Four separate NRC groups were tested:

1. Control group, incubated with Tyrode solution for the entire experimental period;
2. SI/R group, incubated with simulated ischemia buffer for 3 hours hypoxia, followed by 2 hours re-oxygenation;
3. Vehicle group, subjected to 0.2% (v/v) DMSO administration 30 minutes prior to SI/R;
4. SI/R+GSRd group, subjected to GSRd (10 μM) administration 30 minutes prior to SI/R, a dose selected based upon dose-response experiments and previous investigations [5], [24].

### Determination of cardiac function

MI/R-induced cardiac dysfunction was determined by invasive hemodynamic evaluation methods. A micro-catheter was inserted into the left ventricle via the right carotid artery to measure the left ventricular pressure (LVP). ECG and LVP were simultaneously recorded on a polygraph (RM-6200C; Chengdu, Instrument, Chengdu, China). Computer algorithms measured left ventricular systolic pressure (LVSP), left ventricular end-diastolic pressure (LVEDP), first derivative of left ventricular pressure (±d*P*/d*t*_max_), and heart rate (HR) at baseline, after 30 minutes ischemia, and after 1, 2, and 3 hours of reperfusion.

### Determination of myocardial infarct size

After reperfusion conclusion, the coronary artery ligature was retied. 4 mL of 2% Evans blue dye (Shanghai Chemical Reagents, Shanghai, China) was injected into the aorta. Dye was circulated and uniformly distributed, except in the cardiac region previously perfused by the occluded coronary artery (defining the ischemic region or area at risk, AAR). Cardiectomy was rapidly performed. Hearts were frozen at −20°C and sliced into 1-mm sections perpendicular to the base-apex. Slices were incubated in 1% TTC in phosphate buffer at 37°C for 10 minutes (pH 7.4). Morphometric measurements of AAR and infarct area (INF) were performed by image analysis system (Image-Pro plus; Media Cybernetics, Bethesda, MA). Myocardial infarct size was expressed as percentage of infarct area (INF) over total AAR (INF/AAR×100%).

### Determination of in vivo necrosis and cell death

Myocardial cellular damage and necrosis were evaluated by measuring plasma levels lactate dehydrogenase (LDH) and creatine kinase (CK). Blood samples (1mL) were drawn after 3 hours reperfusion. LDH and CK levels were measured in blinded manner by spectrophotometry (DU 640; Beckman Coulter, Brea, CA) in duplicate.

### Determination of myocardial apoptosis

Myocardial apoptosis was determined by a commercially available terminal deoxynucleotidyl nick-end labeling (TUNEL) assay per manufacturer's protocol. TUNEL-positive cardiomyocytes in ischemic myocardium were counted in double-blinded fashion. The percentage of TUNEL-positive cells was determined by dividing the number of positive-staining nuclei by the total number of nuclei in a given field of view (at 200 microscopic magnification).

An additional test was performed to assess myocardial apoptosis with greater specificity. Cardiac caspase-3 activity was determined via caspase-3 colorimetric assay kit (Chemicon, Temecula, CA). In brief, myocardial tissue was homogenized in ice cold lysis buffer for 30 seconds. The homogenates were centrifuged. Supernatants were collected, and protein concentrations were measured by bicinchoninic acid method. To each well of a 96-well plate, supernatant containing 200 µg of protein was loaded and incubated with 25 µg caspase-3 substrate N-acetyl-Asp-Glu-Val- Asp (DEVD)-p-nitroanilide at 37°C for 1.5 hours. The optical density was measured at 405 nm with a SpectraMax-Plus microplate spectrophotometer. Caspase-3 activity was calculated using a standard curve and expressed as fold increase over the mean value of sham MI.

### Determination of cellular viability

Cellular viability was determined by MTT assay. NRCs were distributed into a 96-well plate (density 1×10^5^ cells/well), and pretreated with different GSRd concentrations (0.1–50 µM). After experimental treatment, MTT was added to each well (final concentration 0.5 mg/mL). Plates were incubated for 4 hours at 37°C. Absorbance of blue formazan derivative, indicating viability, was measured at 570 nm via microplate reader (Bio-Rad Laboratories, CA, USA). All measurements were performed in duplicate.

### Determination of in vitro cellular injury

Cellular injury was determined by LDH release. 0.2 mL of culture medium from NRCs post H/R treatment was analyzed by spectrophotometry via commercial assay kit (UV-120-02, Shanghai, China), per manufacturer's protocol. Cellular LDH release was expressed as the percentage of total cell LDH activity. All measurements were performed in duplicate.

### Determination of apoptosis by flow cytometry

The NRC apoptotic ratio was determined by flow cytometry with annexin V-FITC/PI staining per manufacturer's protocol. In brief, NRCs were plated upon a six-well plate, and pretreated with 10 µM GSRd for 30 minutes followed by SI/R treatment. After experimental treatment, cells were collected, washed with calcium-free PBS, and resuspended in binding buffer. Cells were treated with annexin V-FITC and PI, placed in the dark at room temperature for 15 minutes, and analyzed by a Beckton-Dickinson flow cytometer (FACS).

### Measurement of intracellular reactive oxygen species

ROS generation was determined by fluorescent probe DCFH-DA. Cell-permeable non-fluorescent DCFH-DA oxidizes to the highly fluorescent 2,7-dichlorofluorescin in ROS presence. NRCs were plated upon a six-well plate, and pretreated with 10 µM GSRd for 30 minutes followed by SI/R treatment. Cells were harvested by trypsinization. After two PBS washings, 10 µM DCFH-DA was added for 20 minutes at 37°C in the dark. Fluorescence intensity was measured by flow cytometry (Coulter, USA) at excitation wavelength 488 nm, and emission wavelength 525 nm.

### Measurement of mitochondrial membrane potential

Mitochondrial membrane potential (MMP) was evaluated by cationic dye JC-1. In normal cells, JC-1 aggregates in mitochondria, fluorescencing red. In apoptotic cells, JC-1 accrues in the cytosol, as a green fluorescencing monomer. At the experiment's conclusion, 1×10^6^ cells were harvested by trypsinization. After two PBS washings, cells were incubated with JC-1 10 µg/mL for 15 minutes at 37°C in the dark. Cells were harvested, suspended in PBS, and analyzed by flow cytometry.

### Western blot analysis

Whole cell extracts were prepared as follows: Cultured NRCs were washed twice with cold PBS and immersed in lysis buffer (composition: 50 mM HEPES, pH 7.4, 0.1% Chaps, 5 mM DTT, 0.1 mM EDTA, and 0.1% Triton X-100). Cell lysates were centrifuged. Protein concentrations in the supernatants were determined by Bradford Protein Assay Kit (Bio-Rad, CA, USA). Equal samples were loaded onto and separated by 12% SDS-polyacrylamide gel electrophoresis. Proteins were transferred to nylon membranes by electrophoretic transfer system (Bio-Rad). Membranes were blocked in 5% skim milk for 1 hour at room temperature. Incubation with primary antibody commenced overnight at 4°C, followed by secondary antibody conjugated to horseradish peroxidase for 2 hours. Immunoblot was visualized with ChemiDocXRS (Bio-Rad Laboratory, Hercules, CA), and analyzed with LabImage software.

### Statistical analysis

All values are presented as mean±SEM. Differences were evaluated by AVOVA followed by Bonferroni correction for post hoc *t*-test, where appropriate. P values less than 0.05 were considered significant. All statistical tests were performed with GraphPad Prism software, version 5.0 (GraphPad Software, San Diego, CA).

## Results

### Ginsenoside Rd improves rat cardiac function after MI/R

**Figure 2:**
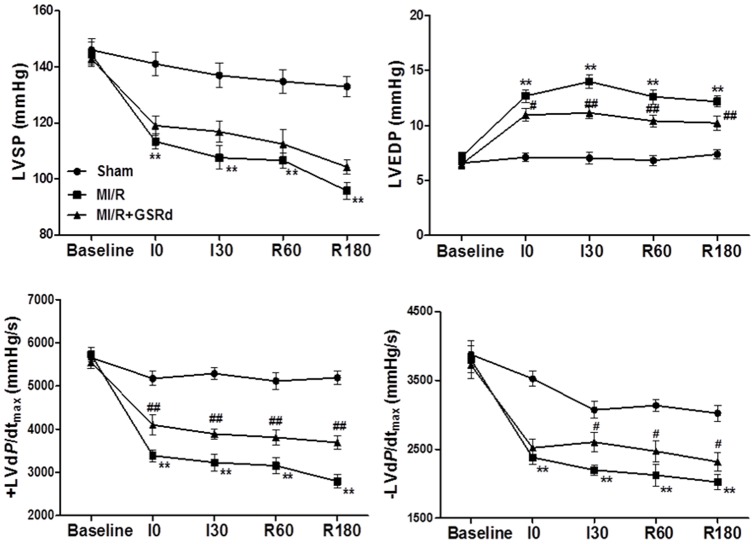
Ginsenoside Rd improves rat cardiac function after 30 minutes ischemia and 3 hours reperfusion. Values presented are mean ± SEM. Abbreviations: LVSP, left ventricular systolic pressure; LVEDP, left ventricular end diastolic pressure; ±LVd*P*/d*t*_max_, the instantaneous first derivation of left ventricle pressure; MI/R, myocardial ischemia/reperfusion (30 minutes/3 hours). n = 8/group. **P<0.01 vs. Sham, ^#^ *P*<0.05, ^##^ *P*<0.01 vs. MI/R.

GSRd had no effects on blood glucose, blood pressure and cardiac function in the absence of MI/R. No significant hemodynamic differences existed between groups at baseline conditions. Additionally, there were no significant differences in heart rate (HR) and mean arterial pressure (MAP) between any groups during MI/R. Pretreatment with GSRd enhanced ± LV*dP*/*dt*_ max_ after 3 hours reperfusion compared to MI/R group (Figure 2). Additionally, GSRd markedly decreased LVEDP post-I/R compared to MI/R group (*P*<0.01). Hemodynamic data support GSRd improved rat cardiac systolic and diastolic function after MI/R.

### Ginsenoside Rd reduced rat myocardial injury (infarct size, necrosis, and apoptosis) post MI/R

**Figure 3:**
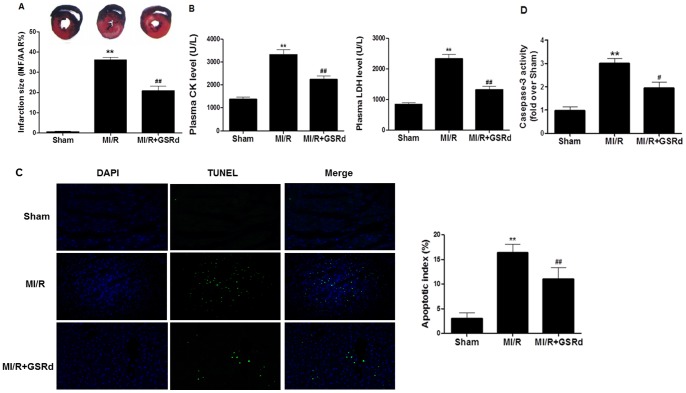
Ginsenoside Rd reduced rat myocardial injury (infarct size, necrosis, and apoptosis) post MI/R. (**A**) Myocardial infarct size in rats subjected to 30 minutes I, followed by 3 hours R. Blue-staining represents non-ischemic tissue, red-staining represents the area at risk, and pale areas indicate infracted regions. Myocardial infarct size (INF) expressed as percentage of area at risk (AAR). (**B**) Plasma creatine kinase (CK) and lactate dehydrogenase (LDH) levels. (**C**) Left: Representative photomicrographs of in situ detection of apoptotic cardiomyocytes by terminal deoxynucleotidyl nick-end labeling (TUNEL) staining in MI/R heart tissue. Green fluorescence indicates TUNEL-positive apoptotic nuclei; blue fluorescence indicates total cardiomyocyte nuclei. Original magnification 200×; Right: Percentage of TUNEL-positive nuclei in heart tissue sections. (**D**) Myocardial caspase-3 activity. All values presented are mean ± SEM. n = 8/group. **P<0.01 vs. Sham, ^#^ *P*<0.05, ^##^ *P*<0.01 vs. MI/R.

Myocardial infarct size and plasma CK and LDH were measured to assess myocardial injury post I/R. Representative AAR and INF images are shown in Figure 3A. No myocardial infarction was observed in sham-group hearts. 30 minutes MI followed by 3 hours R resulted in significant infarction in MI/R group rats compared to sham (36.0%±1.5% versus sham, *P*<0.01). GSRd treatment significantly decreased infarct size (20.9%±2.3% versus 36.0%±1.5% MI/R-group, *P*<0.01). There was no significant difference in AAR between all groups.

Cardiomyocyte necrosis is characterized by cellular content release. To determine whether GSRd attenuated MI/R-induced cardiomyocyte necrosis, plasma CK and LDH levels were measured after reperfusion conclusion. Plasma CK and LDH levels increased to 3,324±228 and 2,327±143U/L respectively in the MI/R-group (Figure 3B). GSRd treatment markedly decreased CK and LDH levels (2,238±160 and 1,320±109 U/L respectively, *P*<0.01) in the MI/R group. These indicators support GSRd decreased in vivo myocardial necrosis post-MI/R.

Apoptosis is the major mechanism of cell death immediately following a short period of ischemia with ensuing reperfusion, and was assessed by two methods, TUNEL staining and caspase-3 activity. As expected, TUNEL-positively staining cells were minimally detected (3.0%±1.2%) in the sham-group (Figure 3C), whereas the MI/R group exhibited a significant number of TUNEL-positively cells (16.3%±1.8%). GSRd pretreatment significantly reduced TUNEL-positively staining cells (11%±2.3%). Myocardial caspase-3 activity is a very specific indicator of cardiomyocyte apoptosis. Consistent with TUNEL results, the MI/R group exhibited significantly increased caspase-3 activity (3.0±0.2 versus 0.9±0.2, *P*<0.01, Figure 3D). GSRd substantially reduced caspase-3 activity compared to the MI/R-group (1.9±0.3 versus 3.0±0.2, *P*<0.05). Together, these data suggest GSRd decreased post-MI/R myocardial apoptosis in vivo.

### Ginsenoside Rd ameliorated in vitro cell death (viability, death, and apoptosis) post SI/R

**Figure 4:**
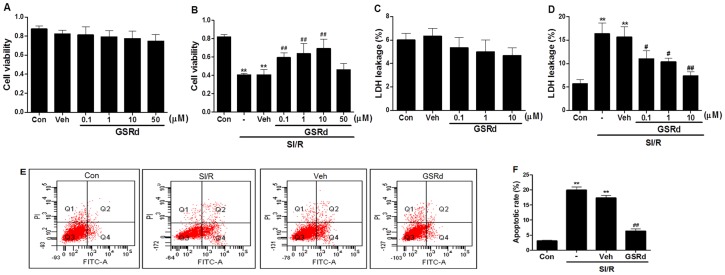
Ginsenoside Rd ameliorated SI/R-induced in vitro cell injury (viability, death, and apoptosis). (**A**) GSRd treatment alone (0.1–50 μM) for 24 hours did not alter NRC viability, suggesting no GSRd-induced toxicity at concentrations up to 10 μM (n = 8; *P>0.05 vs. Control). (**B**) Cellular viability as determined by MTT assay after SI/R (3 hours hypoxia followed by 2 hours reoxygenation). (**C**) Cellular death post GSRd treatment alone for 24 hours as determined by LDH leakage into medium (n = 8; *P>0.05 vs. Control). (**D**) LDH assay in cells administered GSRd (0.1, 1, 10 μM) 30 minutes prior to SI/R. (**E**) SI/R-induced apoptosis as determined by Annexin V-FITC/PI flow cytometry in control and vehicle groups. (**F**) 10 μM GSRd significantly reduced SI/R-induced apoptosis as determined by Annexin V-FITC/PI flow cytometry. All values presented are mean ± SEM. **P<0.01 vs. Control, ^#^ *P*<0.05, ^##^ *P*<0.01 vs. SI/R. These experiments were performed in triplicate with similar results.

To first determine the effects of GSRd alone upon NRCs, cells were treated with varying concentrations of GSRd (0.1–50 µM). GSRd alone at these concentrations for 24 hours was not cytotoxic by MTT and LDH leakage assay (Figure 4A, 4C). Concentration response curves determining cellular viability are shown in Figure 4B. Peak cellular viability was observed at GSRd dose 10 µM.

Cellular viability and LDH leakage are indices of NRCs injury. After being subject to SI/R, cellular viability in the vehicle group was significantly reduced 41%±0.6% compared to control, and LDH leakage increased 16.33%±2.3% compared to control (all *P*<0.01). GSRd (0.1, 1, and 10 µM) markedly reduced SI/R-induced cell death, respectively increasing viability rate to 59%±1.8%, 63%±3.9%, and 69%±3.7% and decreasing LDH leakage to 11%±1.7%, 10.3%±0.9%, and 7.3%±0.9% (*P*<0.01, Figure 4B, 4D). Together, these results indicate GSRd significantly preserved cellular viability post-SI/R injury in a dose-dependent manner (at concentrations up to 10 μM).

Cellular apoptosis was assessed by flow cytometry (Figure 4E). Annexin V/PI double staining demonstrated significant apoptotic increase in vehicle group compared to control post SI/R (19.9%±1.1% versus 3.1%±0.2%, *P*<0.01). 10 µM GSRd markedly decreased apoptosis (6.3%±0.7%, *P*<0.01, Figure 4F). Taken together, these in vitro results support GSRd as a potent cardioprotective agent, in consistent fashion with in vivo data.

### Ginsenoside Rd reduces intracellular ROS generation, increases mitochondrial membrane potential (MMP), and decreases cytochrome c release in NRCs subjected to SI/R

**Figure 5:**
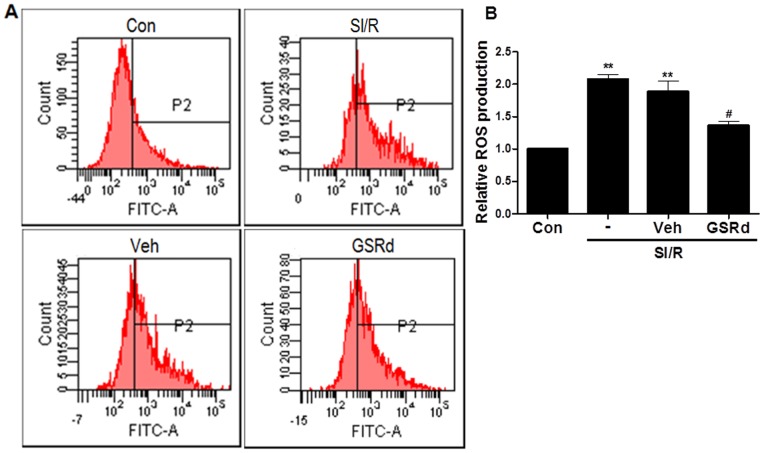
Ginsenoside Rd reduces intracellular ROS generation in NRCs subjected to SI/R. Intracellular ROS accumulation was measured via fluorescence probe DCFH-DA. Fluorescent intensity was determined at excitation wavelength 488 nm and emission wavelength 525 nm via flow cytometry. Values presented are mean ± SEM. **P<0.01 vs. Control, ^#^ *P*<0.05 vs. SI/R. These experiments were performed in triplicate with similar results.

Intracellular ROS levels were assessed by determining DCF fluorescence intensity via flow cytometry. SI/R induced rapidly and significantly increased DCF fluorescence (*P*<0.01, Figure 5A). However, pretreatment of NRCs prior to SI/R significantly decreased DCF fluorescence (*P*<0.05, Figure 5B), suggesting GSRd significantly reduced ROS generation during SI/R in NRCs.

**Figure 6:**
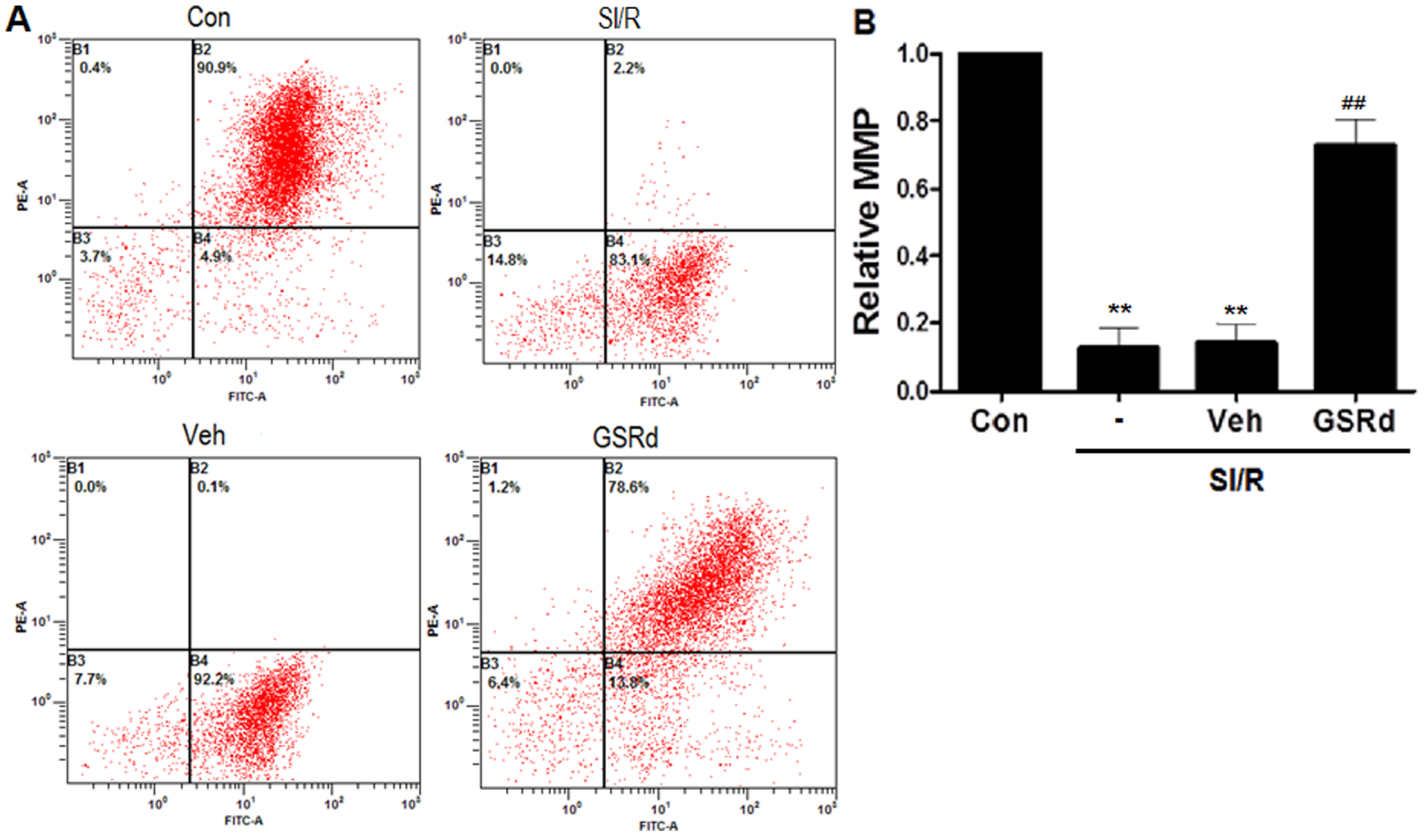
Ginsenoside Rd increases mitochondrial membrane potential (MMP) in NRCs subjected to SI/R. MMP was measured with fluorescent dye JC-1. 10 μM GSRd was administered 30 minutes prior to SI/R. Fluorescent intensity of JC-1 was determined at excitation wavelength 488 nm and emission wavelength 530 nm via flow cytometry. Values presented are mean ± SEM. **P<0.01 vs. Control, ^##^ *P*<0.01 vs. SI/R. These experiments were performed in triplicate with similar results.

**Figure 7:**
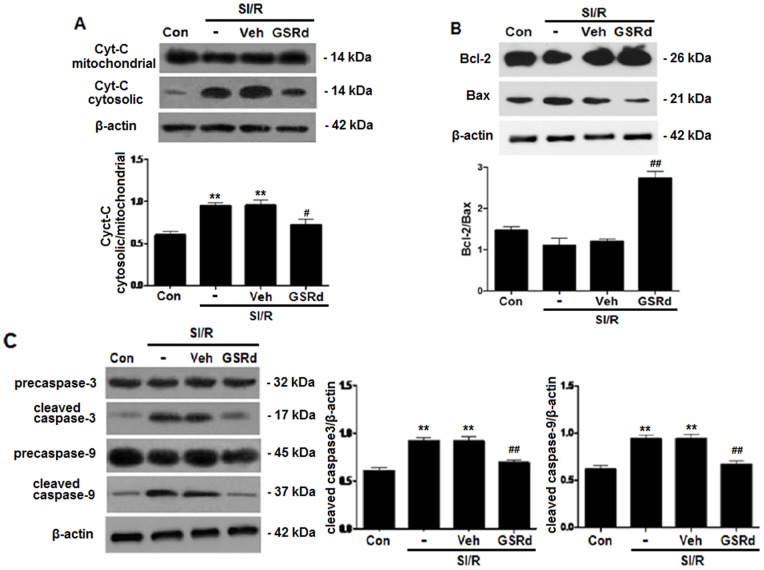
Ginsenoside Rd inhibits mitochondrial-mediated apoptosis in NRCs subjected to SI/R. (**A**) Representative western blot for cytochrome c release. SI/R increased cytosolic translocation of mitochondrial cytochrome c. Densitometric analysis demonstrates 10 μM GSRd inhibited mitochondrial cytochrome c release. (**B**) Representative western blot for Bcl-2 and Bax expression after various experimental treatments. Densitometric analysis demonstrates SI/R reduced the Bcl-2/Bax ratio, but GSRd increased the Bcl-2/Bax ratio. (**C**) Representative western blot for SI/R-induced casepase-3 and caspase-9 activation. Densitometric analysis demonstrates 10 μM GSRd reduced expression of cleaved caspase-9 and caspase-3. All values presented are mean±SEM. n = 6; **P<0.01 vs. Control, ^#^ *P*<0.05, ^##^ *P*<0.01 vs. SI/R.

Mitochondrial membrane potential (MMP) is an important early determinant of the mitochondrial apoptotic pathway. We investigated the effects of GSRd upon MMP and cytochrome c release. MMP detection was performed utilizing JC-1 dye to assess mitochondrial membrane depolarization. NRCs subjected to SI/R exhibited substantially decreased mitochondrial depolarization compared to control (*P*<0.01, Figure 6A). Pretreatment with 10 µM GSRd significantly stabilized the MMP (*P*<0.01, Figure 6B). Mitochondrial depolarization releases several apoptogenic proteins, most notably cytochrome c into the cytosol. Western blot analysis demonstrated SI/R increased mitochondrial cytochrome c release into cytosol, and 10 µM GSRd decreased cytochrome c release (0.9±0.03 versus 0.7±0.02, *P*<0.05, Figure 7A). Together, these results suggest GSRd may attenuate apoptosis by potentially involving the mitochondrial apoptotic pathway.

### Ginsenoside Rd modulates Bcl-2 and Bax expression in NRCs subjected to SI/R

Next, we determine whether GSRd protects against SI/R-induced apoptosis in NRCs by modulating the Bcl-2 family proteins. SI/R treatment decreased Bcl-2 (an anti-apoptotic protein) expression, and increased Bax (a pro-apoptotic protein) expression, decreasing the Bcl-2/Bax ratio (Figure 7B). Pretreating NRCs with 10 µM GSRd prior to SI/R promoted Bcl-2 expression and inhibited Bax expression, increasing the Bcl-2/Bax ratio (Figure 7B).

### Ginsenoside Rd decreases caspase-3 activity in NRCs subjected to SI/R

Caspases regulate cellular apoptosis. Cytochrome c release activates caspase-9, which activates caspase-3. SI/R significantly increased expression of both cleaved caspase-9 and caspase-3, which was attenuated by 10 µM GSRd pretreatment (Figure 7C).

### Ginsenoside Rd increases phosphorylation of Akt and GSK-3β in NRCs subjected to SI/R

**Figure 8:**
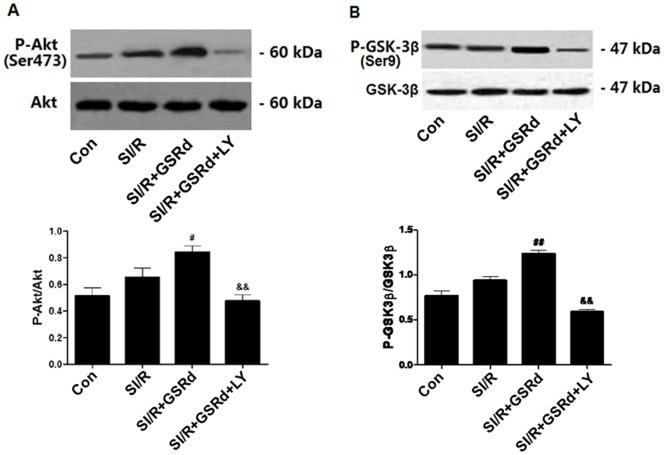
Ginsenoside Rd increases phosphorylation of Akt and GSK-3β in NRCs subjected to SI/R. Densitometric analysis demonstrates GSRd increased the ratio of P-Akt/Akt and P-GSK-3β/GSK-3β, which was significantly blocked by Akt-inhibitor LY294002. Values presented are mean±SEM. n = 6; ^#^ *P*<0.05, ^##^ *P*<0.01 vs. SI/R, ^&&^ *P*<0.01 vs. SI/R+GSRd.

To further investigate the molecular mechanism underlying GSRd-mediated cardioprotection, we determined P-Akt/Akt and P-GSK-3β/GSK-3β in NRCs post SI/R by western blot. There was no significant difference in Akt and GSK-3β expression between treatment groups at baseline (Figure 8). Consistent with previous reports, SI/R alone increased phosphorylation of Akt and GSK-3β. Pretreatment with 10 µM GSRd significantly increased phosphorylation of Akt and GSK-3β (and consequently increased P-Akt/Akt and P-GSK-3β/GSK-3β ratios, *P*<0.01). Pretreatment with PI3K inhibitor LY294002 blocked GSRd-mediated phosphorylation of Akt and GSK-3β.

## Discussion

Several important observations were made in the present study. Firstly, we demonstrate that pretreatment with GSRd attenuated in vivo MI/R injury in a rat model (evidenced by improved cardiac function, reduced infarct size, and reduced myocardial apoptosis after MI/R), and reduced in vitro SI/R injury in cultured NRCs (evidenced by increased cardiomyocyte viability, decreased cardiomyocyte LDH activity, and reduced cardiomyocyte caspase-3 and -9 cleavage). Secondly, we provide the first evidence that GSRd reduces intracellular ROS generation in cardiomyocytes, and inhibits myocardial apoptosis induced by SI/R via the mitochondrial-dependent apoptotic pathway. Finally, we demonstrate Akt/GSK-3β signaling pathway activation significantly contributes to the anti-apoptotic effect of GSRd.

The medical herb ginseng is used worldwide. Ginsenosides, triterpene saponins, are a major ginseng component. More than 40 ginsenosides have been identified. Previous studies demonstrate ginsenosides have significant protective effects in the cardiovascular system [25]–[27]. Wang et al. studied MI/R injury in an in vivo rat model, and reported ginsenoside reduced infarct size and improved resultant myocardial pathologic changes [28]. In a cell culture model, Chen et al. reported panax notoginseng saponins prevented cardiomyocyte apoptosis induced by glucose and oxygen deprivation injury via PI3K/Akt signaling [29]. The ginsenoside GSRd is highly lipophilic, and easily diffuses across biological membranes [5]. Heretofore, its effects against MI/R injury have never been investigated. Ginsenoside Rb1 and Re have been demonstrated to exert direct depressant action upon cardiomyocytes contraction, mediated in part via increased NO production, reducing afterload and improving cardiac pump function [30]. In our current study, GSRd augmented cardiac function, increasing ±LVd*P*/d*t* max and decreasing LVEDP, and reduced intracellular cardiomyocytes ROS generation. Further investigation will be necessary to dissect the mechanisms responsible for such divergent phenomenon. Nevertheless, our study supports in consistent fashion the potential beneficial clinical applications of GSRd.

During physiological conditions, a critical balance exists between free radical production and the endogenous antioxidant system [31], [32]. Pathological conditions such as ischemia and reperfusion tilt the balance in favor of ROS overproduction, increasing oxidative stress, a major apoptotic stimulus. Pharmaceutics inhibiting ROS formation or antagonizing ROS toxicity are cardioprotective against reperfusion injury [12], [33], [34]. In the current study and many others, MI/R injury caused infarction and cardiac dysfunction. SI/R injury in cultured NRCs induced significant cell death. GSRd both limited infarct size and augmented cardiac function in the employed rat MI/R model. GSRd attenuated cellular damage (measured by MTT viability and LDH activity assays) in cultured NRCs subjected to SI/R.

Cardiomyocyte apoptosis is one of the major pathogenic mechanisms underlying MI/R injury [34]. Cumulative evidence suggests that ROS, implicated in reperfusion toxicity, can trigger cardiomyocyte apoptosis via the mitochondrial apoptosis pathway [11], [35], [36]. ROS released during the early phase of myocardial reperfusion strongly oxidizes cardiomyocytes already been damaged by ischemia. Cardiomyocytes are rich in mitochondria, a major endogenous source and susceptible target of ROS damage [37]. Mitochondrial-mediated apoptosis plays an important role in MI/R injury pathogenesis [8]. Under normal conditions, cytochrome c is located within mitochondria. During intracellular ROS overproduction, collapse of the mitochondrial membrane potential (MMP) results in mitochondrial permeability transition pore (mPTP) opening, and rapidly releasing cytochrome c into the cytoplasm. Once released, cytochrome c binds the C- terminal domain of the apoptotic protease activating factor-1 (Apaf-1), inducing a conformation change. The activated Apaf-1/cytochrome c complex promotes caspase activation [38]. Caspases transduce and execute apoptotic signaling [11]. Caspase-3 (of the terminal common apoptotic pathway) is also activated by caspase-9, which is activated by the mitochondria-mediated apoptotic pathway. In the current study, we demonstrate GSRd pretreatment mitigated SI/R-induced intracellular ROS, MMP, and mitochondrial release of cytochrome c into the cytosol, suggesting involvement of the mitochondrial pathway in GSRd-mediated cardioprotection.

The Bcl-2 protein family, compromised of both anti-and pro-apoptotic members, are important mitochondrial regulators during cardiomyocyte apoptosis [12]. Bcl-2 regulates mPTP opening in opposition to Bax, blocking cytochrome c release, inhibiting caspase activity, and decreasing cell apoptosis [39], [40]. Therefore, altering the Bcl-2/Bax ratio influences apoptotic balance. Western blot revealed SI/R significantly decreased the Bcl-2/Bax ratio, an effect reversed by GSRd administration, suggesting GSRd-mediated cardioprotection against SI/R injury may occur partially via modulating Bcl-2/Bax expression.

The serine survival kinase Akt is activated downstream of phosphatidylinositol 3-kinase (PI3K). Activation of PI3K and Akt is cardioprotective against MI/R injury, and prevents cardiomyocyte apoptosis [41], [42]. Akt overexpression in cultured cardiomyocytes preserves mitochondria Bcl-2 levels [18]. Akt exerts its protective effects via phosphorylation of diverse target molecules (such as Bcl-2 family and GSK-3), preserving mitochondrial integrity. A downstream effector of Akt, GSK-3β is phosphorylated at Ser 9 by Akt; phosphorylated GSK-3β attenuates MI/R injury [20]. Phosphorylated GSK-3β suppresses mPTP opening by binding to adenine nucleotide translocase (ANT, one of the mPTP components), thereby reducing the affinity of ANT for cyclophilin D [39]. In the present study, SI/R increased Akt and GSK-3β phosphorylation, consistent with previous reports demonstrating cardioprotective PI3K/Akt signaling in settings such as preconditioning [19], [43]. GSRd pretreatment further augmented Akt and GSK-3β phosphorylation and attenuated cellular apoptosis. The PI3K inhibitor LY294002 partially blocked the effects of GSRd. Together, these results support mechanistic involvement of Akt/GSK-3β signaling pathway in GSRd-mediated anti-apoptotic effect.

Several limitations exist in the current study. Phosphorylation of Akt by GSRd and its inhibition by LY294002 provide strong supportive evidence for the involvement of Akt/GSK-3β in GSRd-induced MI/R protection. However, it is not clear LY294002 completely reverses GSRd's effect upon cellular apoptosis. Additionally, while Akt overexpression preserves mitochondrial Bcl-2 levels [18], but the specific mechanism by which GSRd activates Akt to modulation the Bcl-2/Bax ratio remains unknown, and warrants further investigation.

**Figure 9:**
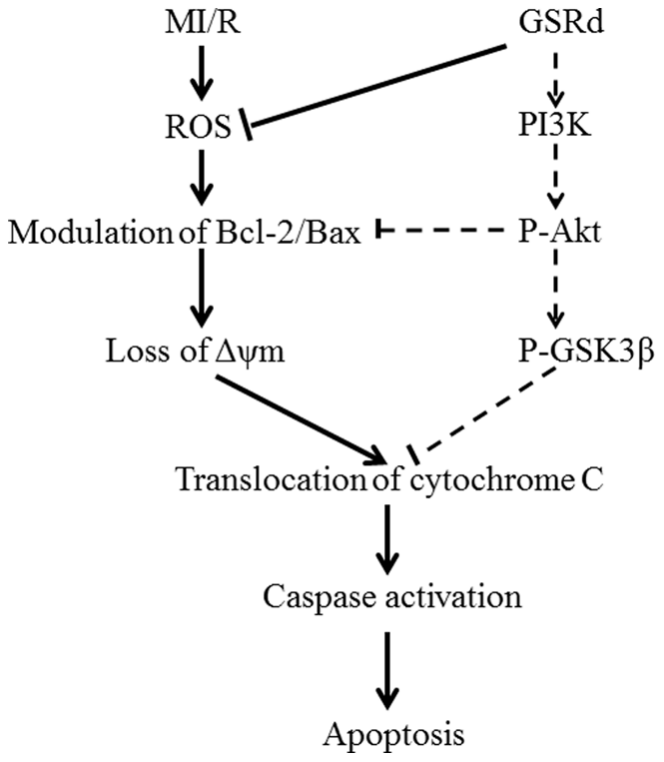
Schematic diagram depicting protective signaling of GSRd in MI/R-induced apoptosis. GSRd inhibits the apoptotic signaling cascades initiated by MI/R-generated ROS. Arrows (→) indicate activation or induction, and segments ending with a (⊢) indicate inhibition/blockade. Solid lines (—) indicate mechanisms strongly supported by the current study, and dotted lines (--) indicate hypothesized connections requiring further investigations.

Taken together, our results demonstrate for the first time that GSRd exerts cardioprotection against myocardial MI/R injury by both reducing intracellular ROS and inhibiting mitochondria-mediated apoptosis. Activation of Akt/GSK-3β signaling is involved in the cardioprotective effect of GSRd (Figure 9). The traditional herbal medicine GSRd may have therapeutic potential attenuating myocardial ischemia/reperfusion injury.
